# The Stress-Response Factor SigH Modulates the Interaction between *Mycobacterium tuberculosis* and Host Phagocytes

**DOI:** 10.1371/journal.pone.0028958

**Published:** 2012-01-03

**Authors:** Noton K. Dutta, Smriti Mehra, Alejandra N. Martinez, Xavier Alvarez, Nicole A. Renner, Lisa A. Morici, Bapi Pahar, Andrew G. MacLean, Andrew A. Lackner, Deepak Kaushal

**Affiliations:** 1 Divisions of Bacteriology and Parasitology, Tulane National Primate Research Center, Covington, Louisiana, United States of America; 2 Divisions of Comparative Pathology, Tulane National Primate Research Center, Covington, Louisiana, United States of America; 3 Department of Microbiology and Immunology, Tulane University School of Medicine, New Orleans, Louisiana, United States of America; Fundació Institut d'Investigació en Ciències de la Salut Germans Trias i Pujol. Universitat Autònoma de Barcelona. CIBERES, Spain

## Abstract

The *Mycobacterium tuberculosis* stress response factor SigH plays a crucial role in modulating the pathogen's response to heat, oxidative-stress, envelope damage and hypoxia. We hypothesized that the lack of this key stress response factor would alter the interaction between the pathogen and its host cells. We compared the interaction of *Mtb*, *Mtb*:Δ-*sig*H and a strain where the mutation had been genetically complemented (*Mtb*: Δ-*sig*H:CO) with primary rhesus macaque bone marrow derived macrophages (Rh-BMDMs). The expression of numerous inducible and homeostatic (CCL) β-chemokines and several apoptotic markers was induced to higher levels in the cells infected with *Mtb*:Δ-*sig*H, relative to *Mtb* or the complemented strain. The differential expression of these genes manifested into functional differences in chemotaxis and apoptosis in cells infected with these two strains. The mutant strain also exhibited reduced late-stage survival in Rh-BMDMs. We hypothesize that the product of one or more SigH-dependent genes may modulate the innate interaction of *Mtb* with host cells, effectively reducing the chemokine-mediated recruitment of immune effector cells, apoptosis of infected monocytes and enhancing the long-term survival and replication of the pathogen in this milieu The significantly higher induction of Prostaglandin Synthetase 2 (PTGS2 or COX2) in Rh-BMDMs infected with *Mtb* relative to *Mtb*: Δ-*sig*H may explain reduced apoptosis in *Mtb*-infected cells, as PTGS2 is known to inhibit p53-dependent apoptosis.The SigH-regulon modulates the innate interaction of *Mtb* with host phagocytes, perhaps as part of a strategy to limit its clearance and prolong its survival. The SigH regulon appears to be required to modulate innate immune responses directed against *Mtb*.

## Introduction

TB, a major cause of infectious disease mortality in humans, is responsible for the death of over 1.7 million people every year [1]. This situation is exacerbated by the emergence of drug-resistant strains of *Mtb* [2], AIDS co-infection [3] and the failure of Bacille Calmette-Guerin (BCG) vaccine [4]. To develop effective anti-TB drugs and vaccines, the mechanisms that help establish a long-term infection by *Mtb* need to be better defined.

*Mtb* uses various strategies to survive within the host, including subversion of phagosome development by secreting effector molecules [5]; the modulation of cytokine expression by secreting immune-modulatory molecules into the cytoplasm of *Mtb*-infected macrophages [6]; the modulation of immune signaling pathways by pumping cyclic AMP using a bacterial adenylate cyclase [7]; and the induction of matrix metalloprotease (MMP9) expression by a secreted mycobacterial protein, - a process essential for the initiation of granuloma formation [8].

The *Mtb* alternate sigma factor, SigH, is induced after heat, redox and acid stress [9], [10], [11], [12] and phagocytosis [13], [14]. An isogenic Δ-*sig*H mutant is attenuated for pathology in a mouse model [9] and for both pathology and bacterial replication in a macaque model [15]. SigH directs the transcription of 31 genes, including those which encode ECF sigma factors SigE and SigB and proteins involved in the maintenance of intrabacterial reducing capacity [9], [10]. Indirectly, SigH may influence the transcription of about one-fifth of the entire *Mtb* genome including those involved in ATP-dependent Clp proteolysis, mammalian cell entry and sulfate utilization [16]. SigH is also a member of the enduring hypoxia response [17] as well as the re-aeration response [18], indicating that members of the extended SigH-regulon may play a role in hypoxia stress defense and perhaps in the reactivation of latent infection. The SigH-dependent Clp proteolysis pathway has recently been implicated in defense against envelope damage [19], [20], [21].

These results suggest that SigH controls the expression of genes critical to the intracellular survival of *Mtb*. In support of this argument, a member of the extended SigH-regulon, Rv2745c (ClgR) is necessary for the successful infection of host phagocytes [22]. We therefore compared the host response to infection with the isogenic *Mtb*:Δ-*sig*H mutant relative to strains containing functional copy of SigH. Towards this study, we chose bone marrow derived macrophages (BMDMs) from rhesus macaques (*Macaca mulatta*). Rhesus macaques have been extensively used as experimental models of natural infection with *Mtb* [15], [23]–[25]. Rhesus macaques accurately represent the various aspects of human TB including acute [15], [23], [24] or latent TB and its reactivation by AIDS co-infection [25].

## Results

### Infection of Rh-BMDMs with *Mtb*, the *Mtb*:Δ-*sig*H mutant and the *Mtb*:Δ-*sig*H:Co complemented strains

Rh-BMDMs were generated by culturing cells from the bone-marrow of several healthy, normal Indian-origin rhesus (*Macaca mulatta*) collected at euthanasia. The macrophage phenotype of the Rh-BMDMs was characterized by multi-label confocal microscopy by staining with anti- Ham56, CD68 and CD163 antibodies (Figure S1) and flow-cytometry as previously described [14].

**Figure 1:**
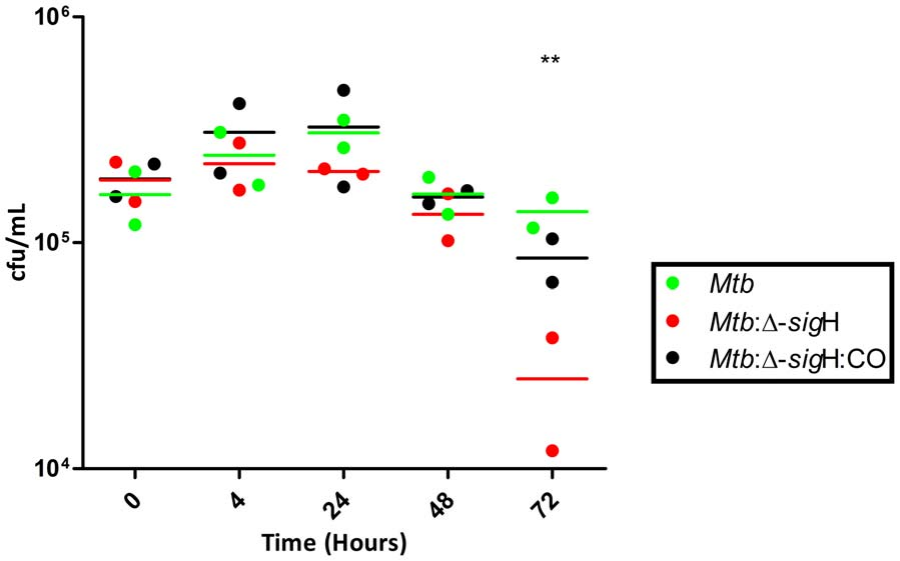
Comparison of infection of Rh-BMDM with *Mtb*, *Mtb*:Δ-*sig*H and *Mtb*:Δ-*sig*H:CO. Growth progression of the infection by *Mtb*, the *Mtb*:Δ-*sig*H mutant and the mutant-complemented strain in Rh-BMDMs over the course of time measured by CFU analysis. The figure shows the arithmetic means ± standard deviations for two biological replicate experiments, with each replicate data comprising of a summarized value from different relevant dilutions and multiple platings.

Rh-BMDMs were infected for four hours with tubercle bacilli at an MOI of 1∶10, as previously described [14]. Samples were then processed for the 0 hr post-infection time-point, or further cultured for the 4, 24, 48 and 72 hours post-infection time-points. All three strains infected Rh-BMDMs to comparable levels, as assessed by the measurement of viable cfu's at 0 hr post-infection (Figure 1). The three strains replicated to comparable extent in Rh-BMDMs initially, increasing about a one-half log in total number during the course of 24 hrs. However, at the late stage (72 hr post-infection), a significantly higher bacterial burden was present in cells infected with *Mtb*, relative to the *Mtb*:Δ-*sig*H mutant. The decline in *Mtb*:Δ-*sig*H cfu's was directly related to the loss of a functional SigH regulon, since the cfu burden in cells infected with the *Mtb*:Δ-*sig*H:CO complemented strain was comparable to the parental *Mtb* strain.

### The global transcriptomic response of Rh-BMDM cells to infection with *Mtb*, *Mtb*:Δ-*sig*H and *Mtb*:Δ-*sig*H:CO

To study the impact of the SigH regulon on host phagocytes, we compared the transcriptome profiles of uninfected Rh-BMDMs to those infected with *Mtb* or the *Mtb*: Δ-*sig*H mutant strain, at all three time-points (cultured 0, 4 or 24 hr after the end of the four hr infection). When transcription in distinct biological replicates of uninfected Rh-BMDMs was compared, very small number of genes (21 at 0 hr, 20 at 4 hr and 76 at 24 hr) showed an alteration in expression by more than 2-fold. These data serve as controls to which the effect of experimental infection with the different strains can be compared.

When biological replicates of Rh-BMDMs infected with *Mtb* were compared to uninfected cells, the expression of 370 genes was induced >2-fold in a statistically significant manner at 0 hrs post-infection time-point, (Table S1-A)and he genes with the greatest induction included CCL20 (5.8-fold), IL8 (3.6-fold), CCL4 (3.6-fold) and CCL7 (2.9-fold).

At the 4 hr time-point, the expression of 180 genes was significantly induced as a result of *Mtb* infection (Table S1-B). Among the genes with the greatest induction at this time-point were CXCL1 (46.9-fold), CXCL3 (31.6-fold), PTGS2 (31.5-fold), CXCL6 (24-fold), CXCR4 (22.8-fold), CXCL2 (10-fold), CCL2 (4-fold) and CCL7 (3.8-fold).

At the 24 hr time-point, the expression of 475 genes was significantly induced as a result of *Mtb* infection (Table S1-C)The list of genes with >2-fold induction following *Mtb* infection at this time-point included PTGS2 (76-fold), CXCL6 (50-fold), CXCL3 (43-fold), CXCL1 (43-fold), CCL2 (38-fold), CCL5 (25-fold), CXCL11 (19-fold), CCL7 (18-fold), and CCL11 (8-fold).

The expression of 277 genes was induced >2-fold in a statistically significant manner by infection with *Mtb*:Δ-*sig*H at the 0 hr time-point(Table S1-D) Some of the genes with the greatest induction included CCL4 (17.6-fold), CXCL2 (10-fold), CCL7 (6.5-fold) and CCL20 (4.3-fold).

At the 4 hr time-point, the expression of 71 genes was significantly induced by more than 2-fold as a result of *Mtb*: Δ-*sig*H infection (Table S1-E). The genes with the greatest induction at this time-point were CXCL1 (45-fold), CXCL6 (18-fold), CXCL3 (13-fold), CCL2 (13-fold) andCXCL2 (10-fold).

At the 24 hr time-point, the expression of 607 genes was significantly induced by more than 2-fold as a result of infection with the mutant (Table S1-F). The list of genes with the greatest induction following *Mtb*:Δ-*sig*H infection at this time-point included CXCL1 (120-fold), PTGS2 (100-fold), CXCL6 (75-fold), CCL2 (53-fold), CXCL3 (40-fold), CXCL1 (43-fold) and CCL2 (38-fold) CCL5 (35-fold).

Following infection with a strain where the mutation in the *sig*H gene was genetically complemented, the expression of 119 genes was induced >2-fold in a statistically significant manner at the 0 hr time-point (Table S1-G) Some of the genes with the greatest induction included CXCL2 (29-fold), CXCL6 (16-fold), CCL7 (11-fold) CXCL1 (6-fold), CCL3 (5-fold) and CCL4 (3-fold).

At the 4 hr time-point, the expression of 189 rhesus genes was significantly induced as a result of infection with the complemented strain (Table S1-H) relative to uninfected cells. The genes with the greatest induction at this time-point were CXCL6 (58-fold), CXCL1 (57-fold), CXCL2 (15-fold), CXCL3 (7-fold), CXCL10 (7-fold), CXCL7 (6-fold) and CCL7 (3-fold).

At the 24 hr time-point, the expression of 463 rhesus genes was significantly induced as a result of infection with the complemented strain (Table S1-I). This list included CXCL1 (30-fold), CXCL6 (29-fold), CXCL3 (10-fold), CCL7 (4-fold) and CCL19 (3-fold).

A number of genes exhibited overlapping induction or repression at different time-points. Therefore we have identified all genes which exhibited such overlapping expression following infection with either *Mtb* or the mutant (Table S2 B).

The expression of several β-chemokine genes was differentially expressed following infection with the mutant, relative to either *Mtb* or the complemented strain. The expression of CCL4 appeared to be significantly higher in cells infected with the *Mtb*:Δ-*sig*H mutant relative to either *Mtb* or the complement, at the 0 hr post-infection time-point, while the expression of CCL2, CCL5 and CCL7 appeared to be significantly higher in cells infected with the mutant relative to *Mtb* or the complemented strain, at 4 hrs post-infection. The expression of CCL13, CCL19 and CXCL1 was significantly higher in cells infected with the *Mtb*:Δ-*sig*H mutant relative to *Mtb* or *Mtb*:Δ-*sig*H:CO at 24 hrs post-infection (Table S1 B).

**Figure 2:**
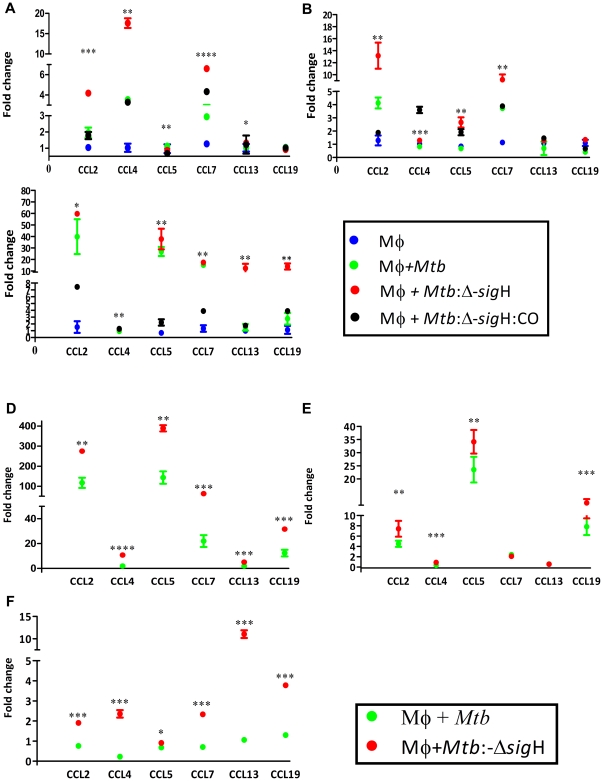
Comparison of chemokine-expression in Rh-BMDM infected with *Mtb or Mtb*: Δ-*sig*H at the RNA level. Expression values are shown for DNA microarray experiments at 0 (A), 4 (B) and 24 (C) hrs. Results for uninfected cells are represented by blue circles, while those for *Mtb*, the mutant and the complemented strain are represented by green, red and black circles, respectively. Values represent responses that were statistically different from the Rh-BMDM (Mφ) responses (p<0.05). Results are representative of two independent experiments. *, p<0.05; **, p<0.01, ***, p<0.001 and ****, p<0.0001. Expression values are also shown for RT-PCR experiments at 0 (D), 4 (E) and 24 (F) hrs. Results for *Mtb* are represented by green open boxes while those for the mutant are represented by red boxes. RT-PCR arrays specific for the chemokines pathway were used to confirm microarray results. GAPDH was used as the invariant housekeeping control for normalization. The figure shows the arithmetic means ± standard deviations for two biological replicates. *, p<0.05; **, p<0.01, ***, p<0.001 and ****, p<0.0001.

We therefore systematically investigated the expression of various chemokines and their receptors. The expression of several β-chemokines was induced at high levels in cells infected with the *Mtb*:Δ-*sig*H mutant. For example, the expression of CCL2, CCL4 and CCL7 was induced to higher levels in cells infected with the *Mtb*:Δ-*sig*H mutant (4.18, 6.23 and 4.3-fold respectively), relative to cells infected with *Mtb* (1.9, 3.5 and 2.9-fold respectively) or *Mtb*:Δ-*sig*H:CO (1.7, 3.3 and 3.7-fold respectively), at the 0 hr post-infection time-point (Figure 2A). Similarly, the expression of CCL2, CCL5 and CCL7 was induced to higher levels in cells infected with the *Mtb*:Δ-*sig*H mutant (13.17, 2.2 and 9.2-fold respectively), relative to cells infected with *Mtb* (4.1, 0.68 and 3.8-fold respectively) or *Mtb*:Δ-*sig*H:CO (1.9, 1.8 and 3.8 respectively), at the 4 hr post-infection time-point (Figure 2B). At the 24 hr post-infection time-point, the average expression of CCL2, CCL5, CCL13 and CCL19 was induced to higher levels in cells infected with the *Mtb*:Δ-*sig*H mutant (59.7, 37.7, 12.3 and 13.9-fold respectively), relative to cells infected with *Mtb* (39.8, 26.9, 1.5 and 2.76-fold respectively) or *Mtb*:Δ-*sig*H:CO (7.4, 2.2, 1.7 and 3.9-fold respectively) (Figure 2C). At this time point, the expression of CCL4 was not significantly different, but was numerically higher in the mutant compared to the other two strains. Clearly, infection with the mutant resulted in a higher expression of both inducible and homeostatic chemokines, in the host phagocytes. These differences are either directly or indirectly governed by the mycobacterial SigH regulon, since genetic complementation of the mutation reversed this differential induction of β-chemokines. Since the microarray results, along with those of cfu analysis overwhelmingly confirmed that the *Mtb*:β-*sig*H:CO complemented strain elicits a transcriptional response and a replication phenotype comparable to the parental *Mtb*, subsequent experiments only addressed the differences between the parental and the mutant strain.

### Confirmation of microarray results at the transcript level by quantitative RT-PCR

In order to confirm the results obtained in microarray studies, we utilized pathway based quantitative PCR arrays (RT_2_ProfilerPCR arrays, SABiosciences), specific for chemokine signaling. The expression of CCL chemokine ligands CCL2, CCL4 and CCL7 was significantly higher in cells infected with the mutant, relative to the cells infected with *Mtb*, immediately following infection at the 0 hr time-point (Figure 2D). The expression of CCL2, CCL5 and CCL7 remained significantly higher than in cells infected with the mutant, relative to the cells infected with *Mtb*, at the 4 hr time-point (Figure 2E). The expression of homeostatic chemokines CCL13 and CCL19 was significantly higher in the cells infected with the mutant, relative to the cells infected with *Mtb*, at the 24 hr time-point (Figure 2F).

### Confirmation of microarray results at the protein level by confocal microscopy

**Figure 3:**
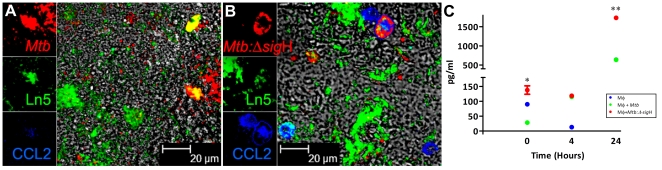
Comparison of the expression of CCL2 in Rh-BMDM infected with *Mtb or Mtb*: Δ-*sig*H at the protein level. Visualization of the production of chemokine CCL2 (blue) in *Mtb* (A) *or Mtb*:Δ-*sig*H (B) (both in red) - exposed (Ln5^+^) macrophages (green) by multilabel confocal microscopy. CCL2 production measured by ELISA (C). Levels of CCL2 were determined in the supernatants of Rh-BMDMs with *Mtb or Mtb*:Δ-sigH by multiplex ELISA. The figure shows the arithmetic means ± standard deviations for three biological replicates. *, p<0.05; **, p<0.01, ***, p<0.001 and ****, p<0.0001.

Since the levels of CCL2 were consistently higher in Rh-BMDMs infected with the *Mtb*:Δ-*sig*H strain in transcript assays, we studied its expression in uninfected and infected cells at different time-points by multilabel confocal microscopy (Fig. 3). TOPRO-3 was used to identify cell nuclei in Rh-BMDM cultures. We found many more cells expressing CCL2 in cultures infected with *Mtb*:Δ-*sig*H for 24 hrs (Fig. 3B), relative to those infected with *Mtb* (Fig. 3A).

### Confirmation of microarray results at the protein level by cytokine/chemokine assay

We performed beadarray assay on the supernatants obtained from Rh-BMDMs infected with either *Mtb* or *Mtb*:Δ-*sig*H at 0, 4 and 24 hr post-infection and compared the results to those obtained from the supernatants from uninfected cells at the same time-points to identify the levels of secreted β-chemokines. CCL2 was found to be present in significantly higher (∼4-fold) levels in the supernatants of Rh-BMDMs infected with *Mtb*:Δ-*sig*H, compared to the supernatants of cells infected with *Mtb*, which harbored CCL2 levels comparable to uninfected supernatants (Figure 3C). Unlike transcript analyses, the difference at the protein level was only apparent at the 24 hr time-point.

### Induction of peripheral blood mononuclear cell (PBMC) migration

**Figure 4:**
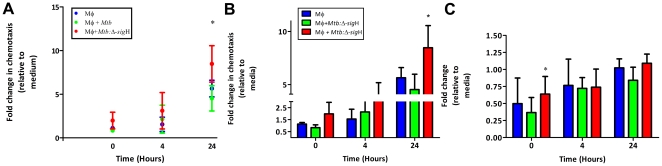
Chemotaxis assay. Migration of PBMCs (A) in response to supernatants from uninfected macrophages (black), macrophages infected with *Mtb* (green), *Mtb*:Δ-*sig*H (red) relative to tissue culture medium. Values are expressed as cells/microscopic field. Similarly, values are also shown for CD14^+^ (B) and CD3^+^ (C) population in the PBMCs.

We designed chemotaxis assays to understand the functional relevance of higher β-chemokine expression in cells infected with the mutant, relative to *Mtb*. Chemotaxis was performed using uninfected, *Mtb*-infected and *Mtb*:Δ-*sig*H-infected cell culture supernatants relative to cell-culture media as a negative control. Autologous, naïve peripheral blood mononuclear cells (PBMCs) were used for chemotaxis assays. PBMCs migrating in response to different stimuli were phenotyped as either macrophages (CD14^+^) or lymphocytes (CD3^+^) by flow cytometry. The total number of cells migrating in response to chemotactic signal from the supernatant of Rh-BMDMs infected for 24 hr with *Mtb*:Δ-*sig*H was significantly higher than the corresponding number of cells from the supernatant of cells infected with *Mtb*. (Figure 4A). The increased number of migrating PBMC could be attributed to CD14^+^ cells (Figure 4B). While the number of CD14^+^ cells that migrated in response to the mutant-conditioned chemotactic signal was higher relative to *Mtb*-conditioned chemotactic signal at the 0 and 4 hr time-points, these differences were not significant. However, a statistically significant (p<0.035), higher number (∼2-fold) of CD14^+^ cells migrated in response to the mutant-conditioned chemotactic signal, relative to the, relative to the *Mtb*-conditioned chemotactic signal at 24 hr. Greater number of CD3^+^ lymphocytes also migrated in response to the mutant-derived chemotactic signal, relative to *Mtb*-derived- or uninfected-chemotactic signal, but the differences were not statistically significant (Figure 4C).

### Differential expression of pro and anti-apoptotic markers in *Mtb*-infected, relative to *Mtb*:Δ-*sig*H-infected Rh-BMDMs

**Figure 5:**
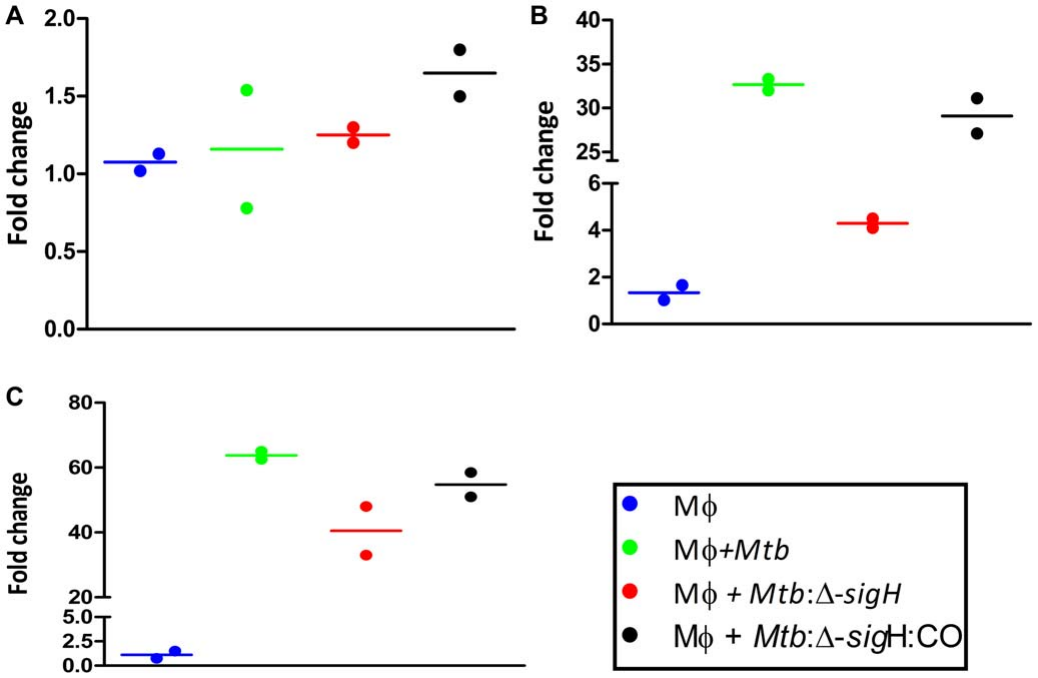
Differential expression of PTGS2 in Rh-BMDMs infected with *Mtb*, relative to those infected with the *Mtb*: Δ-*sig*H mutant. The expression of this gene is shown for 0, 4 and 24 hrs from either uninfected Rh-BMDMs (black open boxes), or cells infected with either *Mtb* (green open boxes) or the mutant (red open boxes). Results are representative of two independent experiments.

The expression of PTGS2 which codes for a prostaglandin dehydrogenase synthase, was highly induced in cells upon infection with *Mtb* (32-fold at 4 hr; 60-fold at 24 hr) and the complement (28-fold at 4 hr; 55-fold at 24 hr), relative to the mutant (4.8-fold at 4 hr; 45-fold at 24 hr) (Fig. 5A, B,C). PTGS2 is involved in the conversion of the pro-apoptotic arachidonic acid into anti-apoptotic and anti-inflammatory prostaglandin substrates. Therefore, we hypothesized that high levels of expression of PTGS2 following *Mtb* expression may allow the pathogen to evade innate immune response by partially inhibiting apoptosis of infected monocytes.

**Figure 6:**
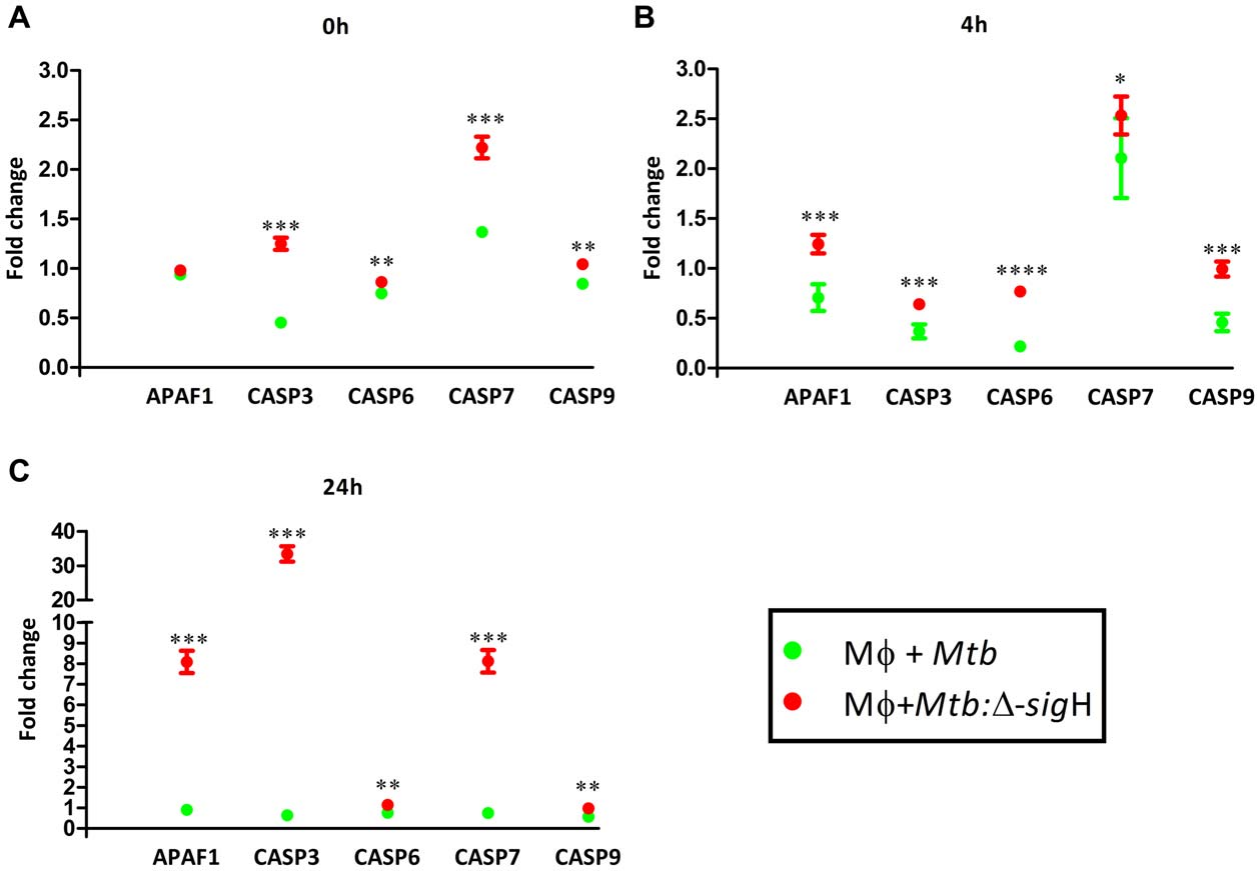
Differential expression of apoptotic genes during infection of Rh-BMDMs with *Mtb*, relative to infection with the *Mtb*:Δ-*sig*H mutant. The temporal expression changes of apoptotic genes as determined at the 24 hr time point by RT-PCR arrays specific for (Caspase dependent) apoptosis,to confirm microarray results at the 0 (A), 4 (B) and 24 (C) hrs. GAPDH was used as the invariant housekeeping control for normalization. The figure shows the arithmetic means ± standard deviations for two biological replicates. *, p<0.05; **, p<0.01, ***, p<0.001 and ****, p<0.0001.

The expression of pro-apoptotic caspase-encoding genes CASP1, CASP3 and CASP6 was indeed induced to a significantly higher level in cells infected with the *Mtb*:Δ-*sig*H, though the levels of induction were not comparable to those observed for β-chemokines. CASP1 levels were induced >4-fold in cells infected with the *Mtb*:Δ-*sig*H, but only ∼1.5-fold in cells infected with *Mtb* and not at all with the complemented strain. CASP3 levels were induced >2-fold in cells infected with the *Mtb*:Δ-*sig*H, but remained unchanged in cells infected with either *Mtb* or the complemented strain. CASP6 levels were induced >3-fold in cells infected with the *Mtb*:Δ-*sig*H, but only ∼2-fold in cells infected with *Mtb* and the complemented strain. We therefore analyzed the expression of numerous pro-apoptotic genes in infected cells using RT-PCR. The expression of CASP3, CASP6, CASP7 and CASP9 was induced to higher levels in cells infected with the *Mtb*:Δ-*sig*H, relative to cells infected with *Mtb*, at the 0 hr time-point (Figure 6A). The expression of APAF1, CASP3, CASP6, CASP7 and CASP9 genes was induced to higher levels in cells infected with the *Mtb*:Δ-*sig*H, relative to cells infected with *Mtb*, at both the 4 (Figure 6B) and 24 (Figure 6C) hr time-points.

### Confocal microscopy based TUNEL to assay for macrophage apoptosis

**Figure 7:**
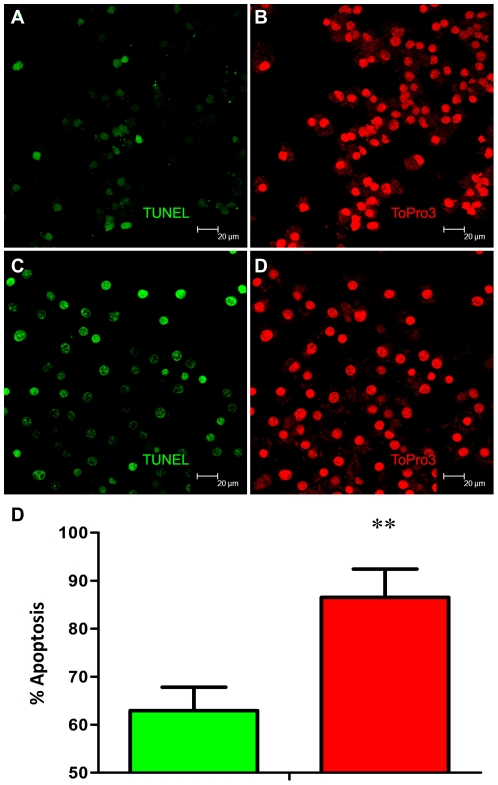
Functional validation of differential expression of apoptotic genes in Rh-BMDM infected with *Mtb or Mtb*: Δ-sigH by TUNEL assay. Apoptotic macrophages show TUNEL-positive nuclei stained green . Representative results are shown for cells infected with Mtb (A) and the mutant (C) at the 24-hr post-infection time-point The total number of cells (stained with TOPRO-3 for nuclear DNA in red) can be seen for Mtb (B) as well as the mutant (D). Magnification is to a bar scale. Percentage of macrophage undergoing apoptosis after 24 hours of exposure to Mtb or Mtb: Δ-sigH as quantified by in situ TUNEL assays (E).

Since the expression of various components of the apoptotic pathway was induced to higher levels in Rh-BMDMs infected with *Mtb*:Δ-*sig*H relative to those infected with *Mtb*, we examined the extent of apoptosis in both types of infected cells, relative to un-infected cells, as a function of time, using a confocal microscopy based TUNEL assay (Figure 7). Significantly more apoptosis, as measured by TUNEL positivity, was observed for cells infected with *Mtb*:Δ-*sig*H at 24 hrs (Figure 7B), relative to cells infected with *Mtb* (Figure 7A). Quantitative data from counting positive cells from ten fields in each section exhibited a statistically significant difference between from *Mtb*:Δ-*sig*H and *Mtb* (Figure 7C).

## Discussion

In spite of the host mounting an extensive response against it, *Mtb* is able to survive in human tissues. It is therefore considered a highly successful pathogen. It has been hypothesized that *Mtb* modulates the host's immune response, thus interfering with clearance of infection and allowing a persistent or latent infection to develop [26]. *Mtb* is known to interfere with host signaling pathways activated by IFNγ [27], [28], [29], [30], [31] including through the induction of negative regulators of immune signaling e.g. Suppressors of Cytokine Signaling [32], repression of pro-inflammatory chemokines [33] and the down-regulation of the IFNγ receptor [34].

Apart from inhibiting the IFNγ pathway, *Mtb* also attempts to alter other innate and adaptive immune responses [7] [8]. Recently, it has been shown that SigE, an *Mtb* extracytoplasmic sigma factor related to SigH allows the pathogen to modulate host response by partially repressing chemokine secretion [35].

Chemokines facilitate the recruitment of effector immune cells essential for the formation of granulomas. Therefore it is not surprising that numerous chemokine ligands and receptors are expressed in response to *Mtb* infection. These include the several α- (C-X-C-family) [36], as well as β- (C-C-family) chemokines [37].

Our data indicates that the induction of β-chemokines is modulated by infection with *Mtb*. The increased expression of CCL2, CCL4, CCL5, CCL7, CCL13 and CCL19 in cells infected with the Δ-*sig*H mutant and the reversal of this phenomenon in a strain where the mutation was complemented, indicates that a SigH-dependent *Mtb* factor interacts with the host innate immune system to bring about this modulation. CCL2 and CCL7 are the primary chemoattractant signal for monocytes and macrophages, while CCL3 is thought to be secreted by inflamed macrophages to attract polymorphonuclear leukocytes. CCL4 is also produced by inflamed macrophages and attracts NK cells. CCL5 is important for protection against *Mtb* infections. CCL13 and CCL19 are homeostatic chemokines with an established role in the organization of the TB granuloma [38]. A partial downregulation of the β-chemokine signal may be advantageous to *Mtb*, as it can reduce the rate of the recruitment of activated immune cells to the site of infection which have the potential of limiting the infection. Alteration in α/β chemokine ratio may result in increased neutrophil rather than monocyte recruitment, leading to a poor control of infection [39]. This is likely to be evolutionarily more beneficial to the pathogen as the higher number of granulomatous lesions may directly correlate with a greater chance of reactivation.

Apoptosis is an important component of the innate immune response against infectious agents [40]. Therefore, a pathogen that can persist in host tissues for long periods of time, likely modulates these responses [41]. *Mtb* has been shown to possess such anti-apoptotic mechanisms [42], [43], [44]. Furthermore, Pan et al [45] have recently shown that the susceptibility of different genotypic mouse strains to *Mtb* infection is linked to their ability to cause apoptosis. Clearly, the inhibition of macrophage apoptosis may be beneficial to *Mtb*, allowing it to persist for a longer time by evading bactericidal activity [46] and through bypassing CD8 T-cell priming [47], [48].

Based on these results, we hypothesize that the SigH-dependent regulon modulates the interaction of host innate immune responses with *Mtb*. The expression of *Mtb* SigH is induced in a wide-variety of stress conditions such as heat-shock [11], [49]; redox stress [9], [10], low-pH [12], phagocytosis [13], [14], etc. Expression of SigH causes the downstream induction of >700 *Mtb* genes due to, including the SigE, SigB and the Clp proteolysis regulons [15]. One or more products of this large regulon may interact with the host immune system. We propose that the pro-inflammatory signal following *Mtb* infection is counter-balanced by the activation of PTGS2 (COX2) [50]. COX2 not only reduces the cellular concentration of the pro-apoptotic arachidonic acid, but has been can directly interfere with p53 mediated apoptosis [51], [52]. A recent report suggests that a member of the *Mtb* PE_PGRS conserved gene family induces the expression of COX2 in response to stress [53]. The increased production of COX2 by *Mtb*-infected phagocytes may be responsible for the modulation in apoptosis. A mutation in the SigH regulon is able to unmask this effect.

Our results may potentially impact the field of TB vaccines development. *Mtb* mutant strains such as Δ-*sig*H or Δ-*nuo*G [43], which induce a higher degree of apoptosis, are likely to generate a more potent cellular immune response. In fact BCG strains lacking one or more *sig*H alleles have been shown to induce greater immune response [54], leading to a hypothesis that the ineffectiveness of BCG vaccines may stem from the increased immune suppression mediated by multiple copies of *sig*H and the resulting increase in antioxidant levels [55]. *Mtb* mutants that promote apoptosis may be more efficacious vaccine vehicles [56].

## Materials and Methods

### Ethics Statement

The Tulane National Primate Research Center (TNPRC) facilities are accredited by the American Association for Accreditation of Laboratory Animal Care and licensed by the US Department of Agriculture. All animals are routinely cared for according to the guidelines prescribed by the NIH Guide to Laboratory Animal Care. The TNPRC conducts all research in accordance with the recommendations of the Weatherall report - “The use of non-human primates in research”. Bone-marrows were collected from NHPs scheduled for regular necropsy. Animals were humanely euthanized by the veterinary staff at the TNPRC in accordance with endpoint policies. Euthanasia was not performed by any of the authors of this manuscript, nor was any of the animals assigned to the principal investigator's study. Samples were only collected post-euthanasia. All procedures related to *Mtb* were approved by the Tulane Institutional Bio safety Committee (IBC).

### Preparation and maintenance of Rh-BMDM

2 mL bone-marrow from Indian origin rhesus (*Macaca mulatta*) free of SIV, STLV, Type D retrovirus and Herpes B virus, was centrifuged and suspended in phosphate-buffered saline (PBS) (Gibco). The suspension was filtered and the filtrate consisting of enriched macrophages was plated. Rh-BMDMs were grown in Iscove's modified Dulbecco's medium (IMDM, Mediatech) supplemented with 10% heat-inactivated fetal bovine serum (Gibco) and 1% penicillin/streptomycin (BioWhittaker). Rh-BMDMs were extracted using trypsin/versene, and resuspended at a final concentration of 10^6^/ml. The phenotype of the culture cells was >95% pure macrophage type by immunophenotyping, by the measurement of CD14, CD86, HLA-DR, and CD11b [17].

### Infection of Rh-BMDMs with *Mtb* the *Mtb*: Δ-sigH (mutant) and the *Mtb*:Δ-*sig*H:CO (complemented) strains

*Mtb* CDC1551, its isogenic Δ-*sig*H mutant and a complemented strain were cultured as described [9]. Bacterial cultures and infection of Rh-BMDMs at an MOI of 1∶10 have been previously described. After 4 h, the cells were washed extensively to remove extracellular bacilli and either used for the 0 hr post-infection time-point, cultured for another 4, 24 or 72 hours. Intracellular bacteria were obtained by lysing the cells with sterile PBS containing 0.1% saponin (Sigma). The released bacilli were serially diluted in PBS containing 0.01% Tween-80 (Merck) and plated on Middlebrook 7H10/OADC agar in triplicate. CFUs were counted after 21 days of incubation at 37°C.

### DNA Microarray experiments and analysis

0, 4 and 24 hr post-infection, host transcripts extracted from ∼5×10^6^ cells using the RNeasy kit (Qiagen), were used to profile the expression of rhesus genome using 4×44 k rhesus microarrays (Agilent) [24]. Control samples (uninfected) were labeled with Cy3, whereas experimental samples (infected with *Mtb*, *Mtb*:Δ-*sig*H or *Mtb*:Δ-*sig*H:Co) were labeled with Cy5. The analysis methods have previously been described in detail [24]. Raw and normalized array results from our experiments have been deposited into the Gene Expression Omnibus. Genes whose expression changed by at least 2-fold (*P*<0.05) were considered differentially expressed in a significant manner.

### Confirmation of transcriptome results by RT-PCR

To validate transcriptome results, the human RT_2_ProfilerPCR arrays for Chemokines/Receptors (PAHS-022C-12) and Apoptosis (PAHS-012A-12) (SA Biosciences) were employed, using an ABI 7900 real-time PCR machine (Applied Biosystems, Foster City, CA). The data for biological duplicates were analyzed using the SABiosciences PCR Array Data Analysis Software (http://www.sabiosciences.com/pcr/arrayanalysis.php).

### Confirmation of transcriptome results by multiplex cytokine analysis

Supernatants collected from cells infected with *Mtb* or *Mtb*:Δ-*sig*H or in medium alone for 0, 4 and 24 hours were used for quantification of secreted cytokines and chemokines using the NHP cytokine 23 milliplex™ map kit (Millipore) according to manufacturer's directions).

### Chemotaxis assays

Experiments were carried out in 24-well plates with inserts, in a humidified incubator at 37°C with 5%CO_2_. Control or infected BMDM conditioned medium or supernatants (600 µl) were added to lower compartments of 3 µm pore multiwall inserts filters (Becton Dickinson). 100 µl freshly isolated PBMCs at 10^6^/ml from normal macaques were added to the upper compartment and allowed to transmigrate for 2 hrs. The number and phenotype of migrated cells was determined by staining with CD11b (Immunotech), CD14 (BD Pharmingen) and CD3 (Biosource) and performing flow-cytometry as previously described [57]. Input samples were similarly stained.

### Immunofluorescence

After removal of the supernatant, uninfected or infected Rh-BMDMs were harvested using trypsin, Brefeldin-A blocked, washed in PBS, and fixed with 4% paraformaldehyde in PBS, pH 7.0 (USB) for 5–10 minutes for the detection of chemokines. The following dilutions of these antibodies were used: CD163–1∶50 (Serotec), Ham56–1∶20 (Dako)-, CCL2–1∶10 (BD) and Ln5–1∶50 (Zymed). *Mtb* and CD68 were stained as previously described [58].

### TUNEL assay

TUNEL assay was performed using in situ cell death detection kit, fluorescein (Roche Applied Science), according to the manufacturer's instructions. The percentage of apoptotic cells from ten fields were counted from each section (more than 500 cells in all cases). All counts were made by viewing slides under a fixed magnification of 63× (corresponding to an area of 0.05 mm^2^) using a TCS-SP2 confocal microscope (Leica Microsystems).

### Statistical tests

Statistical significance was determined using a Mann-Whitney test in Graphpad Prism), except for microarray results where a t-test script within Spotfire DecisionSite/S^+^ Array Analyzer was used.

## Supporting Information

Figure S1 Identification of the macrophage phenotype of Rh-BMDMs by immunofluorescence. The image shows that a large majority of cultured Rh-BMDMs stain positive for differentiated macrophage marker CD163 (in green). Several of these cells are also double positive for the macrophage marker Ham56 (in red). Inset shows a cell staining positive for both Ham56 (red) and macrophage marker CD68 (green).
(JPG)

Table S1 (A) This table contains a comprehensive list of Rh-BMDM genes with a higher expression in a statistically significant manner, in early (0 h) infection with *Mtb*, relative to uninfected cells. (B) This table contains a comprehensive list of Rh-BMDM genes with a higher expression in a statistically significant manner, in early (4 h) infection with *Mtb*, relative to uninfected cells. (C)This table contains a comprehensive list of Rh-BMDM genes with a higher expression in a statistically significant manner, in early (24 h) infection with *Mtb*, relative to uninfected cells. (D) This table contains a comprehensive list of Rh-BMDM genes with a higher expression in a statistically significant manner, in early (0 h) infection with *Mtb*: Δ-*sig*H mutant, relative to uninfected cells. (E) This table contains a comprehensive list of Rh-BMDM genes with a higher expression in a statistically significant manner, in early (4 h) infection with *Mtb*: Δ-*sig*H mutant, relative to uninfected cells. (F) This table contains a comprehensive list of Rh-BMDM genes with a higher expression in a statistically significant manner, in early (24 h) infection with *Mtb*: Δ-*sig*H mutant, relative to uninfected cells. (G) This table contains a comprehensive list of Rh-BMDM genes with a higher expression in a statistically significant manner, in early (0 h) infection with *Mtb*:Δ-*sig*H:CO complemented strain, relative to uninfected cells. (H) This table contains a comprehensive list of Rh-BMDM genes with a higher expression in a statistically significant manner, in early (4 h) infection with *Mtb*:Δ-*sig*H:CO complemented strain, relative to uninfected cells. (I) This table contains a comprehensive list of Rh-BMDM genes with a higher expression in a statistically significant manner, in early (24 h) infection with *Mtb*:Δ-*sig*H:CO complemented strain, relative to uninfected cells.
(XLSX)

Table S2 Overlap between different microarray data sets. Data are presented here as fold change ratio of *Mtb*:mutant. Regulation: ↑, up; ↓, down. One symbol (↑/↓) indicates the regulation is same for both the strains and two symbols (↑↓/↓↑) indicate the first for *Mtb* and the second for the mutant strain, respectively.
(XLSX)
