# Supplementary figures and images for: The Stress-Response Factor SigH Modulates the Interaction between Mycobacterium tuberculosis and Host Phagocytes
Source: PLoS One. 2012 Jan 3;7(1):e28958. doi: 10.1371/journal.pone.0028958 (PMC3250399; doi:10.1371/journal.pone.0028958)

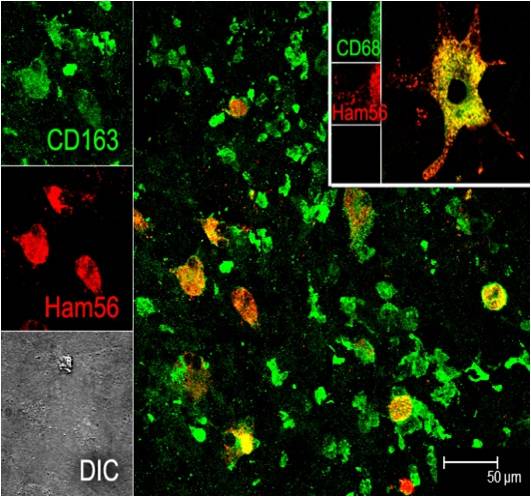

Supplement: Figure S1 — Identification of the macrophage phenotype of Rh-BMDMs by immunofluorescence. The image shows that a large majority of cultured Rh-BMDMs stain positive for differentiated macrophage marker CD163 (in green). Several of these cells are also double positive for the macrophage marker Ham56 (in red). Inset shows a cell staining positive for both Ham56 (red) and macrophage marker CD68 (green). (JPG) [file pone.0028958.s001.jpg]
